# An Experimental Study on Anchoring Effect of Consumers’ Price Judgment Based on Consumers’ Experiencing Scenes

**DOI:** 10.3389/fpsyg.2022.794135

**Published:** 2022-02-08

**Authors:** Yi Zong, Xiaojie Guo

**Affiliations:** ^1^Postgraduate Office, Tianjin University of Commerce, Tianjin, China; ^2^School of Management, Tianjin University of Commerce, Tianjin, China

**Keywords:** consumer experience, decision-making bias, anchoring effect, consumers’ price judgment, experience marketing

## Abstract

Consumers are prone to cognitive biases in decision-making due to the impact of time restrictions, specific environment, and project inducements in the process of experience. Compared with traditional marketing scenarios, it is easy to bias decision makers due to the existence of anchor information. Research on anchoring effect focuses on psychology, economics, law, and medicine instead of the price judgment of consumers. This article uses experimental research to explore the existence and influencing factors of anchoring effect when consumers judge and estimate the price of a product in experiencing scenes. In this article, the hypothesis is that anchoring effect exists and is influenced by factors including anchor value, gender, emotion, personality, knowledge and skill, time pressure, early warning indication, cognitive need, and self-confidence level under external and internal anchor conditions. Subjects judged and estimated different prices after product experience through the design of different decision-making scenarios of external (high anchors and low anchors) and internal anchors, and finally, the anchoring index (AI) and the mean skew index were used to calculate the anchoring effect. The experimental results showed that consumers were affected by anchoring effect when making price judgment in experiencing scenes. In addition to the factors of time pressure and self-confidence level, gender, personality, knowledge, and skill all had a significant influence on anchoring effect under external anchor conditions. Finally, this article provides advice for enterprise marketing planners including setting reasonable anchor values, highlighting the design of experiencing scenes, and developing differentiation strategies.

## Introduction

Back in the 1950s, Abbott (1956) first mentioned the concept of “customer experience,” arguing that consumers really expect not the product, but the experience in the process of consumer products. This experience is the customer’s subject perception and value of everything experienced through the whole consumption process (Gan, 2019). Under the condition of modern market economy, customer experience not only determines customers’ own perception and satisfaction after consumption, but also can spread outward at the speed of geometric series through modern marketing environments, thus affecting the consumption decisions and production or service performance of others (Tong et al., 2020).

Experience marketing comes into being compared with traditional marketing methods. Experience marketing puts more emphasis on customer demand, which can improve the brand value and customer loyalty by improving the experience of customers during consumption (Yang, 2015). Experience marketing encourages consumers to use and feel the products and pay attention to the interactive communication with consumers (Wang et al., 2021). Customers often get a consumer experience, and they share the experience with others, creating product experience value. In the sharing economy, social value is a new driver of consumer contentment and continuance intention (Meilhan, 2019). Experience value is not created by customers alone and must be built in the process of good interaction with others, covering a range of internal and external experience value, such as functional experience, emotional value, social experience, and content experience (Chen and Zhu, 2019; Li, 2019).

This article innovatively puts forward that the key to experience marketing is how to create more experience value for customers by creating the perfect experience of consumers in addition to encouraging consumers to take the initiative to pay a high premium for their products and services. Compared with the solidification of traditional marketing scenes, the experience scene emphasizes more on creating value for consumers through “feeling.” It is usually difficult to accurately measure the price, quality, etc., hence, experience relies on consumers to make price judgment on products and services through sensory impression. However, in the process of product experience, businesses tend to play a dominant position. As a master of a lot of product information and its set experience environment, experience intuitive perception, experience of interaction, and social factors will largely influence the consumers’ cognition of products (Yang, 2017). This will likely lead consumers to make irrational judgments on decision-making problems, leading to decision-making deviation.

Previous studies confirmed that one of the causes of decision bias from behavioral psychology was the presence of anchor effects (Tversky and Kahneman, 1974). The final decision is biased toward the most initial information value, and it is one of the most robust cognitive biases in human decisions (Furnham and Boo, 2011). When people make judgments about something, they are often influenced by first impression or first information, like an anchor into the deep sea, fixing the minds of people and keeping the decision results away from reality (Kahneman and Tversky, 1979).

This study believes that compared with the traditional marketing scene, the “feeling” of the experience scene is often solidified as the impressive first information due to the restrictive (short) of the field experience time, the specific environment, and the induction of the service, so it is likely to produce a certain degree of anchoring effect. This study tries to explore whether the consumer price judgment decision in the experience scenario is affected by the anchor effect and the influence of the consumer price judgment.

Through literature sorting, the existing research on the factors influencing price judgment mostly focuses on product and use value, supply and demand relationship, consumer psychology, product quality (brand and reputation), and other factors (Yu et al., 2017). Domestic research on anchoring effect is concentrated in psychology (Chen et al., 2014), economics (Yao et al., 2021), law (Song, 2019), and medicine (Yan et al., 2021). Foreign research on anchoring effect focuses on research paradigms, theoretical interpretation, and overall influencing factors (Czerwonka, 2017; Yoon and Fong, 2019; Li et al., 2021). As the anchoring effect is one of the reasons for the decision bias, the research in the consumer price judgment is obviously insufficient, especially in literature studies about the relationship between the product price and the anchoring effect in the customer experience scenarios.

Therefore, the innovation point of this research is to study the existence and influence factors of consumer anchor effect in the process of price judgment by setting an experiment to help enterprise marketing planners to make reasonable “anchor strategy,” to improve the perception of consumer experience value, and to stimulate their willingness to buy.

## Theoretical Background Analysis and Research Hypotheses

### Customer Experience Research

Due to the intuitive feeling, participation interaction, and demand creation characteristics of customer experience, customers pay more and more attention to the process and experience of value generation and not just the product itself. Domestic and foreign scholars define the concept of customer experience from different perspectives and dimensions, such as Gentile et al. (2007) who defines it as a reaction from the customer based on the interaction between products or services and enterprise. Luo and Chen (2019), on the other hand, suggest a positive correlation between customer experience perception and purchase intention, and claims that enterprises which want to improve customer engagement need to improve customer experience.

In terms of the experience dimension, Toffler (1970) divides the experience into simulated environment experience and real environment experience starting from authenticity. Real environmental experience will bring real profits and losses to customers, and the simulated situational experience will make customers feel fresh. The experimental approach to use scenario simulation is based on this feature. As early as the late 1980s, the SERVQUAL model for the quality of service tool was proposed. The model measures customer experience perception from five dimensions of visibility, reliability, responsiveness, credibility, and safety. Su et al. (2019) evaluated the customer experience from six aspects of convenient and fast experience, environmental comfort experience, commodity and service experience, price experience, credit guarantee experience, and characteristic innovation experience. This article also plans to draw on the above dimensions to enable customers to judge the price from the product features, design concept, service experience, and other dimensions.

### Decision Deviation and Anchoring Effect

To sum up, during the product experience process, businesses are often in the dominant position and consumers are in a weak position. It is particularly prominent in the Internet era that public figures and social media influencers favorably shape the boost of product awareness. A large number of social media users rely on the suggestions of well-known public figures and refer to their shopping decisions, thus tending to choose the online purchase (Bizzi and Labban, 2019). Details from public figures, social media influencers, and individuals whom they personally know determine the shopping decisions of consumers (Bratu, 2019; Cooley and Parks-Yancy, 2019). Rather than complete information-based mastery of information, it is easy for consumers to make irrational judgments due to estimated decision-making problems due to incomplete information mastery, resulting in decision-making bias. Decision-making deviation is a gap between human subjective will and objective reality, and this deviation is inevitable.

Kahneman and Tversky (1979) confirmed from the perspective of behavioral psychology that people are not always rational when making judgment estimates of future uncertain things, causing a serious deviation between the final judgment estimate and the actual value. The anchor effect is one of them. Furnham and Boo (2011) argued that the anchoring effect is biased toward the most initial information value and that the decision bias is particularly strong. The rise and development of anchor effect in psychology was first discovered by Tversky and Kahneman (1974) in the “The Wheel of luck” experiment, which reached the conclusion of decision makers due to the presence of anchor information. They believe that the anchor effect is when people estimate the problem in an uncertain situation using the first presented information. The initial value holds the thinking of the decision maker like an anchor, and makes the final judgment favor a judgment bias in the initial information. Subsequently, different scholars respectively confirmed the existence form of the anchor effect from different subject fields. The related studies of Yuan (2013), Shen et al. (2016), Feng and Zheng (2019), and Von Hecker et al. (2019) have all taken this concept as the standard, confirming that the anchor effect has a wide and difficult influence to eliminate in the decision-making process of people.

Anchor value is a necessary factor for the anchor effect. Epley and Gilovich (2001) divided the anchor values into external and internal anchors according to their sources. External anchor is an external reference value directly provided by others and is divided into high anchor and low anchor. On the other hand, internal anchor is a reference standard generated by the individual without any information from the outside world based on existing experience and information clues. The anchor effect is divided into traditional, basic, and spontaneous anchor effects. The traditional and basic anchor effects are from outside. The anchor values are from the own experience and inner beliefs and are not affected by the external environment. Therefore, starting from the anchor paradigm, three decision contexts were set up in the later paper: external anchor high anchor group, external anchor low anchor group, and internal anchor group.

### Research Status of the Anchoring Effect of Consumer Price Judgment

The anchoring effect is universal, and it is widely found in financial management, asset evaluation, legal judgment, psychology, medicine, and other research fields. Part 1 of this article mentioned that the anchoring effect has not much literature in the field of price judgment, but in the recent 2 years, this field has also received more and more attention in theory and practice, especially in the stock financial market.

Holst et al. (2015) found that exogenous anchor values could lead to negative adjustment in public auctions and were affected by previous bids as an “anchor.” Gergaud et al. (2017) analyzed the data on grape and vineyard prices in Champagne, France and concluded that anchor had a great impact on grape and vineyard prices and that the anchor effect did not decrease over time. Research by Disli et al. (2020) found that the cross-section data of share price information is closely related to the value of the company. By predicting stock prices with company value as an “anchor,” the results show that higher stock prices are consistent with a higher valuation. Koçaş and Dogerlioglu-Demir (2020) placed random values in specific environments: anchored information, alphanumeric brand names, and inspired numbers, and studies found that random values affect the price perception of consumers. Initial values extracted from a particular environment serve as anchors in subsequent judgments.

Zheng (2015) confirmed by experimental methods that in the two-step question experiment, there are significant anchoring effects during the price judgment estimation process. Irrespective anchors have a large impact on the estimates of subsequent prices. High anchors result in higher estimates and low anchors result in lower estimates. Zhang and Dong (2019) proved that there is a significant anchor effect in the accounting career judgment and decision-making. The whole decision-making process will be affected by the anchoring information. Chu (2020) found that the listed company used information asymmetry to directly anchor the cost of financing and purchase of equity in the stock price, which guide external investors to be optimistic about the merger. Finally, the major shareholders of the listed company plundered the interests of the minority shareholders by reducing their holdings. Zhang and Liu (2021) further found that there is an “anchor effect” in the open-end fund investors of China, and the “anchor effect” led to the redemption vision in the fund market. Lower investor attention, higher uncertainty, higher information complexity, and lower decision-making costs will all exacerbate the anchoring effect of investors.

Through the above discussion, the anchor effect does have an impact on the price. However, most of the existing studies in the field of consumer price judgment are based on the traditional marketing situation, and few scholars mention the experience marketing scene. Nowadays, the traditional marketing model has been gradually eliminated by most enterprises. The new models of experiential marketing, relationship marketing, database marketing, and other network marketing have gradually entered the business stage. In these special fields, except for some research on the relationship between the price and the anchor effect in the network marketing situation, the research in other situations is still a blank (Feng and Zheng, 2019).

### Research Hypothesis

In conclusion, the anchor effect is validated in studies of decision problems in many fields, demonstrating from different perspectives that it is ubiquitous and difficult to eliminate the cause of decision bias. This study aims at the blank points of previous research, grasps the experience marketing scene, and boldly puts forward relevant assumptions. The purpose is to study whether consumers make biased price estimates because of the existence of experience perception, that is, to verify whether the anchor effect exists. If this is true, it will deeply explore the influencing factors causing the anchor effect, enrich the price judgment theory, and the anchor effect theory theoretically, and provide a basis for enterprise marketers to better grasp the consumer decision-making behavior from the perspective of psychology and neuroscience [Mirică (Dumitrescu), 2019; Mircică, 2020].

#### The Existence of the Anchoring Effect

As mentioned above, in this specific situation of customer experience, when consumers make unfamiliar product price decisions due to the emphasis on “experience intuitive perception,” they are easily affected by the set experience environment of merchants. They, therefore, experience interactive field factors. This leads to consumer scarcity of higher cognitive resources, which will produce more anchor adjustments and thinking processing to make up for cognitive scarcity. According to 2.2, the anchor value can be used as the reference for decision making, which is necessary for the anchor effect. Depending on the source, the anchor values are divided into external and internal anchors. External anchor is a reference value for the decision maker, which is divided into external high anchor and external low anchor. High anchor is given reference value higher than actual value, and low anchor is given reference value lower than actual value. The internal anchor is when the outside world provides no information, and the decision makers estimate the problem according to their own experience and subjective judgment (Wang, 2013). Therefore, according to this paradigm, this article adopts the following view: the anchor effect is divided into the external anchor effect and the internal anchor effect (Xu, 2015).

In the customer experience scenario, consumers experience the product according to experience price decision perception, because consumers are often in a weak passive side. Whether under external anchor or internal anchor condition, their price decision problem is an uncertain decision under incomplete information conditions. Due to knowledge and experience temptation will appear initial information curing its thinking and judgment phenomenon, anchor point is more obvious. Therefore, the hypothesis is presented as follows:

H1: Consumers are affected by anchoring effect when making price judgment in a consumer experiencing scene.

#### Influencing Factors of Anchoring Effect

According to previous studies, influencing factors of anchoring effect include anchoring information characteristic, gender, age, emotion, personality, expert knowledge and skill, time pressure, early warning indication, cognitive need, inner belief, and self-confidence level. To explore the influencing factors of anchoring effect in a consumer experiencing scene, the following hypothesis was put forward:

##### Anchor Value

When consumers judge whether the current price is reasonable or not in a traditional marketing scenario, they will take the past price as the reference value (Briesch et al., 1997). That is, a high anchor causes a high price estimate, and a low anchor causes a low price estimate (Simonson and Drolet, 2004). Customer experience, a new marketing mode, is influenced by perception, thinking, behaviors, emotion and so on, but the role of price still exists. According to the above analysis, consumers will refer to information provided by the outside world when judging unfamiliar products in an experiencing scene due to the existence of anchoring effect. Thus, the following hypothesis was put forward:

H2: Consumers use anchor value as a benchmark and make biased price estimation in an experiencing scene.

##### Gender

Zou et al. (2007) believed that gender is an important factor influencing decision-making in uncertain situations. Compared with task-oriented men, society-oriented women are often influenced by emotions and expressions. Lauriola and Levin (2001) found that gender differences lead to different cognitive styles, judgment criteria, and anchoring effect. Women are more likely to be influenced by the representation information when making decisions. In an experiencing scene, consumers are affected by subjective emotional factors when judging the price, and experience perception affects the judgment of consumers on the cost-effectiveness of products. Thus, the following hypotheses were put forward:

H3: Gender affects anchoring effect when consumers make price judgment in an experiencing scene;

H3a: Female consumers are more strongly influenced by anchoring effect than male consumers.

##### Emotion

Emotion is divided into positive emotions and negative emotions. During the 1970s, psychological researchers found that the emotional state of individuals had an impact on their cognitive process of things (Zheng et al., 2012). Schwarz (2002) and Englich and Soder (2009) found that decision makers rely heavily on the representation information of things when judging questions in a positive emotional state. In addition, decision makers conduct more thought processing of the information and make more rational decisions when judging questions in a negative emotional state. When consumers make decisions in an experiencing scene, they are likely to be affected by their emotional states. Thus, the following hypotheses were put forward:

H4: The emotions of consumers affect anchoring effect when they make price judgment in experiencing scenes.

H4a: Consumers in a positive emotional state are more strongly influenced by anchoring effect than consumers in a negative emotional state.

##### Personality

Individual differences directly determine the process and the result of decision-making. Personality is the most important characteristic in individual differences. “Big Five Personality Model” is the most influential personality research paradigm at present, which includes open, conscientious, extrovert, agreeable, and neurotic personalities (Eroglu and Croxton, 2010). Lauriola and Levin (2001) also demonstrated with experiments that different personalities lead to very different decisions.

In experiencing scenes, decision-making of consumers on a question is largely based on their perceived value. Personality difference is an important factor affecting behavioral decision-making and perceived value (Ge and Li, 2020). Typically, highly neurotic individuals are more likely to be influenced by low emotion when making decisions, and they have frequent emotional fluctuations (Chauvin et al., 2010). Highly open and highly extroverted individuals usually make more processing of the anchoring information due to their lack of ability to think independently and intellectually in decision-making (Mcelroy and Dowd, 2007). Highly agreeable individuals get along well with others, while less agreeable individuals are suspicious and do not take risks (Pour and Taheri, 2019). Highly conscientious individuals can better control and manage their emotions (Weng et al., 2016) while less conscientious individuals tend to be careless and adventurous. Thus, the following hypotheses were put forward:

H5: Personalities affect anchoring effect when consumers make price judgment in experiencing scenes;

H5a: Consumers of a low neurotic personality are more strongly influenced by anchoring effect than consumers of a high neurotic personality;

H5b: Highly extrovert consumers are more strongly influenced by anchoring effect than less extrovert consumers;

H5c: Highly open consumers are more strongly influenced by anchoring effect than less open consumers;

H5d: Less agreeable consumers are more strongly influenced by anchoring effect than highly agreeable consumers;

H5e: Less conscientious consumers are more strongly influenced by anchoring effect than highly conscientious consumers.

##### Expert Knowledge and Skill

Expert knowledge and skill refer to the familiarity of consumers with a product. The price judgment of consumers on products is largely dependent on the accumulation of their own experience and knowledge. Consumers who have purchasing experience or are more familiar with the question to be judged make more rational price estimates, and they rely on their own experience with little information processing, while consumers without background knowledge are more likely to be influenced by information like prices, cognition degrees, emotions, etc. when making purchase decisions (Epley and Gilovich, 2006; Reitsma-van Rooijen and Daamen, 2006). Thus, the following hypotheses were put forward:

H6: Knowledge and skill affects anchoring effect when consumers make price judgment in experiencing scenes;

H6a: Consumers with less knowledge and skill are more strongly influenced by anchoring effect than consumers with more knowledge and skill.

##### Time Pressure

Time pressure is the pressure the subjects experience when they make decisions on a given question within the specified time. Mussweiler and Englich (2005) find that the decisions of subjects were influenced by a rapidly presented potential anchor in the anchoring effect study. A decision maker without time pressure will carefully analyze and distinguish the anchor value, while a decision maker with time pressure is more inclined to adopt a heuristic strategy. Time limitation of consumer experience affects the perception of consumers on a product, and the time frame of decision-making also affects the judgment of results of consumers. Thus, the following hypothesis was put forward:

H7: Time pressure affects anchoring effect when consumers make price judgment in experiencing scenes.

##### Early Warning Indication

Epley and Gilovich (2001, 2006) gave the subjects early warning indication in the experiment, and then they found that the decision made by subjects who received early warning indication was closer to the actual value. This was because the decision makers (subjects) deliberately avoided the influence of anchoring effect and made judgment based on objective facts and their own experience. Experience marketing is to select target customers by enhancing experience perception, and the fuzziness of experience process is high. Thus, the following hypothesis was put forward:

H8: Early warning indication affects anchoring effect when consumers make price judgment in experiencing scenes.

##### Cognitive Need

Cognitive need is the willingness of an individual to proactively think when judging unknown matters. Cacioppo and Petty (1982) believed that people with high cognitive need tend to explore, think, and reflect information truthfully when they learn about the development and change of things, while people with low cognitive need are more dependent on their past theoretical research and make decisions which are more heuristic. Thus, the following hypotheses were put forward:

H9: Cognitive need affects anchoring effect when consumers make price judgment in experiencing scenes;

H9a: Consumers with low cognitive need are more strongly influenced by anchoring effect than consumers with high cognitive need.

##### Self-Confidence Level

Self-confidence level is the degree to which individuals are convinced of their own judgment. Generally speaking, consumers with a low self-confidence level feel difficult to make up their minds and are more apt to choose elusion. They make more logical reasoning and theoretical thinking in decision-making, while consumers with a high self-confidence level are affected by inadequate adjustment mechanism and thus, are prone to show excessive confidence. Therefore, the following hypotheses were put forward:

H10: Self-confidence level affects anchoring effect when consumers make price judgment in experiencing scenes;

H10a: Consumers with a high self-confidence level are more strongly influenced by anchoring effect than consumers with a low self-confidence level.

## Experimental Design

### Research Method Selection and Experience Scene Design

Research method selection: According to the requirements of the customer experience scene, the research method combining the scenario simulation experiment method, role playing method, interview, and questionnaire survey method is selected. The situational simulation experimental method is also known as the “field test method.” By arranging the subjects in a simulated and realistic working environment, they can conduct a series of tests on their respective tasks, and finally evaluate the test results.

**FIGURE 1:**
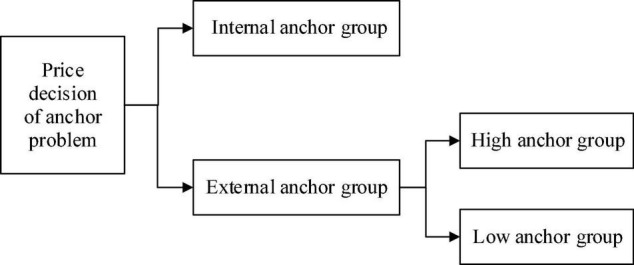
Experimental design group testing of the price judgment of consumers affected by anchoring effect.

Through the design of experiment group (high anchor group and low anchor group) and control group (internal anchor group), the subject (ordinary consumer), in a specific environment of three experience decision situations, is allowed to experience the function and characteristics of the product Figure 1. They are then asked to process and answer the various price judgment problems that may appear in this process. Meanwhile, the role-playing experiment in social psychology is done to complete the experimental design. The main test should explain the experience situation to the subject before the real scenario simulation. It will ask the subject to imagine the role of the ordinary consumer. Finally, the basic information survey and the preliminary experiment rationality evaluation were conducted through the site interview and questionnaire survey to ensure the smooth progress of the formal test.

Experience scene selection: in order to truly reflect the experience marketing environment, “subject perception,” “value transfer,” and other key characteristics, this article selected high-tech products as experimental products. This is because the function of high-tech products is relatively complex and must be used to be able to feel the value of the product or service, thereby making the customer experience very prominent and important. Huawei VR Glass, smart body fat scale 2 Pro, and Free Buds 3 wireless Bluetooth headset were selected as the test experience products. The reasons for the choice of products were as follows: first, the three products were emerging technological products, which would bring feeling of freshness to the subjects; second, consumers without purchasing experience had little knowledge and experience about these products, while customers with purchasing experience could better understand the configuration of these products, so it was less difficult and fuzzy for the subjects to evaluate their own knowledge about these products; third, the three products had no obvious gender audience, so the measurement of gender variables was more objective. After multiple negotiation, the scene simulation experience location is located in a Huawei authorized experience store in Chengdu. All participants acted as ordinary consumers, so that they were able to answer the corresponding price questions according to their experience and their own past experience after experiencing the above products.

### Experimental Design

#### Experimental Subjects and Experimental Products

The experiment was divided into the pre-experiment and the formal experiment. The purpose of the pre-experiment was to find the loopholes of the experimental design in order to optimize the formal experimental flowchart. Thirty subjects selected for the pre-experiment and 240 subjects selected for the formal experiment were all ordinary consumers of an authorized Huawei experience store in a city. These subjects were 18 years old and could make their independent judgment of prices. The basic information of the subjects (sex, age, education, and occupation) met the normal distribution (Table 1). Huawei VR Glass, smart body fat weighing 2 Pro, and Free Buds 3 wireless Bluetooth headset are selected as the test experience products.

**TABLE 1:** Basic information of the subjects.

	Basic Information	Number of People	Proportion
Gender	Male	124	51.67%
	Female	116	48.33%
Age	18–30	70	29.17%
	30–40	61	25.42%
	40–50	53	22.08%
	Over 50	56	23.33%
Occupation	State organs and public institutions	50	20.83%
	Technician	71	29.58%
	Business and service personnel	69	28.75%
	College Students	50	20.83%
Education	Senior high school (technical secondary school) and below	87	36.25%
	Junior college	66	27.50%
	Undergraduate	57	23.75%
	Master degree or above	30	12.50%
			

#### Variable Control and Variable Measurement

##### Control of Anchor Value

Anchor value of the high anchor group was about twice the actual price of a product, and anchor value of the low anchor group was about half the actual price of a product. Anchor value of the internal anchor group was the median of its effective estimated value.

##### Control of Time Pressure

The subjects were divided into three groups: the high anchor group, the low anchor group, and the internal anchor group. In each group, subjects with time pressure and subjects without time pressure were evenly distributed. Subjects without time pressure were not strictly restricted by time when answering questions. Subjects with time pressure had to complete the product experience and make judgment on questions within the specified time. The instruction was “Please complete the questionnaire about product experience and price decision-making within the given time.” A timer was used to remind the subjects of the time, and questionnaires not completed within the given time would be invalid. Time limit for answering questions was based on the test results of the pre-experiment.

##### Control of Early Warning Indication

In the experiment, early warning indication was conducted in the form of an early warning instruction. In each group, subjects were divided into those with early warning indication and those without early warning indication. Before the subjects with early warning indication made price estimates, the early warning indication was presented as follows: ‘This experiment is about anchoring effect. You may be affected by relevant or irrelevant information when making decisions on the price after your product experience. Please make reasonable decisions to avoid judgment bias.’ The subjects without early warning indication received no hint, and their corresponding questionnaires did not include early warning indication.

##### Measurement of Anchoring Effect

In this article, the Anchoring Index (AI) proposed by Jacowitz and Kahneman (1995) and the Mean Skew Index proposed by Epley and Gilovich (2006) were used to measure the anchoring effect.

*Anchoring Index*. Anchoring index (AI) was suitable for the measurement of external anchors (high external anchors and low external anchors). It referred to the ratio of the difference between the estimated value and the difference between the anchoring value under two different anchoring values. The higher the anchoring index was, the greater the anchoring effect would be.


(1) $$A\,I=\frac{\begin{array}{c} \mathrm{Median}\,\mathrm{of}\,\mathrm{estimated}\,\mathrm{value}\,\mathrm{of}\,\mathrm{high}\,\mathrm{anchor}\,\mathrm{group}-\mathrm{Median}\\ \mathrm{of}\,\mathrm{estimated}\,\mathrm{value}\,\mathrm{of}\,\mathrm{low}\,\mathrm{anchor}\,\mathrm{group} \end{array}}{\mathrm{High}\,\mathrm{anchor}\,\mathrm{value}-\mathrm{Low}\,\mathrm{anchor}\,\mathrm{value}}$$

The range of AI was generally 0–1. If AI = 0, it indicated that there was no anchoring effect; if AI = 1, it indicated that there was a strong anchoring effect; if AI > 1, it indicated that there was an extremely significant anchoring effect. In addition, AI could measure the anchoring effect of the high anchor group and the low anchor group, respectively.


(2) $$A\,I_{H\,i\,g\,h}=\frac{\begin{array}{c} \mathrm{Median}\,\mathrm{of}\,\mathrm{estimated}\,\mathrm{value}\,\mathrm{of}\,\mathrm{high}\,\mathrm{anchor}\,\mathrm{group}-\mathrm{Median}\\ \mathrm{of}\,\mathrm{estimated}\,\mathrm{value}\,\mathrm{of}\,\mathrm{control}\,\mathrm{group} \end{array}}{\mathrm{High}\,\mathrm{anchor}\,\mathrm{value}-\mathrm{Median}\,\mathrm{of}\,\mathrm{estimated}\,\mathrm{value}\,\mathrm{of}\,\mathrm{control}\,\mathrm{group}}$$

(3) $$A\,I_{l\,o\,w}=\frac{\begin{array}{c} \mathrm{Median}\,\mathrm{of}\,\mathrm{estimated}\,\mathrm{value}\,\mathrm{of}\,\mathrm{low}\,\mathrm{anchor}\,\mathrm{group}-\mathrm{Median}\\ \mathrm{of}\,\mathrm{estimated}\,\mathrm{value}\,\mathrm{of}\,\mathrm{control}\,\mathrm{group} \end{array}}{\mathrm{Low}\,\mathrm{anchor}\,\mathrm{value}-\mathrm{Median}\,\mathrm{of}\,\mathrm{estimated}\,\mathrm{value}\,\mathrm{of}\,\mathrm{control}\,\mathrm{group}}$$

In Formula (2) and (3), the internal anchor group was used as the control group (the subjects estimated the prices of decision-making questions without any information from the outside world). The high anchor value in Formula (2) was the estimate of 85% percentile in the estimation distribution of the control group. The low anchor value in Formula (3) was the estimate of 15% percentile in the estimation distribution of the control group.

*Mean Skew Index*. The mean skew index was used for the study of internal anchors. The calculation formula is as follows:


(4) $$M\,e\,a\,n-S\,k\,e\,w-I\,n\,d\,e\,x=\frac{\begin{array}{c} \mathrm{Mean}\,\mathrm{of}\,\mathrm{estimated}\,\mathrm{value}-\mathrm{Estimated}\\ \mathrm{boundary}\,\mathrm{value}\,\mathrm{close}\,\mathrm{to}\,\mathrm{the}\,\mathrm{internal}\,\mathrm{anchor} \end{array}}{\begin{array}{c} \mathrm{Estimated}\,\mathrm{boundary}\,\mathrm{value}\,\mathrm{close}\,\mathrm{to}\,\mathrm{the}\,\mathrm{internal}\\ \mathrm{anchor}-\mathrm{Estimated}\,\mathrm{boundary}\,\mathrm{valueaway}\,\mathrm{from}\,\mathrm{the}\\ \mathrm{internal}\,\mathrm{anchor} \end{array}}$$

The mean skew index was generally less than or equal to 0.5. If the Mean Skew Index was 0.5, it indicated that the estimated value was exactly at the center of estimated range, and there was no anchoring effect. If the Mean Skew Index was less than 0.5, it indicated that there was anchoring effect, and the smaller the value was, the stronger the anchoring effect would be.

##### Measurement of Emotion

Emotion induction is usually used to explore the psychological reaction in different emotional states. Referring to the procedure of Gross screening and evaluating materials of film clips, the emotion induction effect was evaluated by subjective reporting with video materials that were expected to induce positive and negative emotions (Gross and Levenson, 1995). The effect was scored (scoring range 0–8) by the Likert nine-point scale. The higher the score was, the stronger the emotion of the subjects would be.

##### Measurement of Expert Knowledge and Skill

Expert knowledge and skill were measured by a scale. The scale of each product contained five questions, and each question was measured by the Likert five-point scale. The higher the score was, the more agreed the subjects would be on the point. Take the first product as an example. The five questions were: (1) I am familiar with the common functions of the product; (2) I am familiar with the working principle of the product; (3) I am satisfied with the cost-performance of the product; (4) I am familiar with the material of the product; and (5) I can choose the product rationally. The reliability and validity tests of the scale showed that the Cronbach’s α of the three products was 0.776, 0.809, and 0.943 respectively, all of which were greater than 0.65, indicating that the scale had high reliability. According to the validity analysis, the scale Kaiser-Meyer-Olkin (KMO) of the three products was 0.907, 0.825, and 0.936, respectively, and the cumulative total variance of the overall measurement questions was 81.54%, which was far greater than the minimum acceptance level of 60%, indicating that the scale had high validity. Therefore, the scale was used as a material for the formal experiment.

##### Measurement of Big Five Personality

Big Five Personality was measured by the Big Five Inventory (BFI) scale. The BFI scale measured five dimensions with adjective phrases by the five-level scoring method. Specifically, “1” meant “strongly disagree” and “5” meant “strongly agree.” There were only a few questions and the average Cronbach’s α was above 0.80, indicating that the scale had high reliability and was easy to operate (John and Srivastava, 1999).

##### Measurement of Cognitive Need

This study selected the cognition need scale (NFC) containing 18 questions developed by Cacioppo et al. (1984) as the measuring tool for cognitive need. The questions were sentences about personality statements. The Cronbach’s α was 0.86 and the scale KMO was 0.90, indicating that the scale had high reliability and validity (Cacioppo et al., 1984). The questions were evaluated by the Likert five-point scale.

##### Measurement of Self-Confidence Level

The self-confidence level was measured by the Rosenberg scale. The scale had high validity and was easy to operate, which consisted of five positive scoring questions and five negative scoring questions. The subjects evaluated the questions by the four-level scoring method.

## Experimental Process

### Pre-experiment

#### Determination of Emotion-Inducing Materials

Studies have shown that the more familiar a subject is with the emotional material, the more likely their emotion will be induced. According to the basic principles of moderate video duration, easiness for understanding, and effective induction determined after consulting literature, this article finally selected the videos of *Hands up!* And *Old Summer Palace* which could induce positive emotions. Then, the video clip software was used to clip the part that could obviously induce emotions. The average video duration was 139 s.

#### Exploring the Influence of Time Pressure on Anchoring Effect

During the experiment, there was no time control on variable measurement, and time pressure was only exerted on the experience of the subjects and their price judgment. Through the statistics of time in each step, the average time for each subject in their experience was 1,740 and 94 s in their price judgment. In this article, the experience time was set as 870 s, and the time for their price judgment was 47 s, both of which were half of the average used time. Subjects who had no time pressure were not subject to time constraints.

Finally, the pre-experiment determined the emotion-inducing materials and time, which saved time for the formal experiment and found out the loopholes of the design. On the basis of the pre-experiment, the formal experiment was carried out.

### Formal Experiment

#### Price Decision-Making Scenarios

To ensure the comprehensiveness of the measurement in the experiencing scene, three decision-making scenarios, i.e., high anchor experiment, low anchor experiment, and internal anchor experiment were set up to measure the external and internal anchoring effect. Details of the three price decision-making scenarios are given in Table 2.

**TABLE 2:** Price decision-making scenarios of consumers.

Experimental scenario	Price decision-making 1	Price decision-making 2
High anchor group	Q1: In your opinion, whether the price of VR glasses is higher than 6 000 yuan or lower Q2: In your opinion, whether the price of smart body fat scale is higher than 1 000 yuan or lower Q3: In your opinion, whether the price of wireless Bluetooth headset is higher than 2 000 yuan or lower	Price that you think may be reasonable
Low anchor group	Q1: In your opinion, whether the price of VR glasses is higher than 1 500 yuan or lower Q2: In your opinion, whether the price of smart body fat scale is higher than 250 yuan or lower Q3: In your opinion, whether the price of wireless Bluetooth headset is higher than 500 yuan or lower	Price that you think may be reasonable
Internal anchor group	Q1: What is the reasonable price for VR glasses in your opinion Q2: What is the reasonable price for smart body fat scale in your opinion Q3: What is the reasonable price for wireless Bluetooth headset in your opinion	Please write down the rationale for your answer

#### Experimental Material Selection

Based on the process and results of the pre-experiment, the formal experiment optimized the used materials, including the basic information questionnaires, emotion-inducing materials (*Hands Up!* And *Old Summer Palace*), emotion determination tables, knowledge and skill measurement tables, BFI scales, NFC, self-confidence level scales, and price decision-making questionnaires.

**FIGURE 2:**
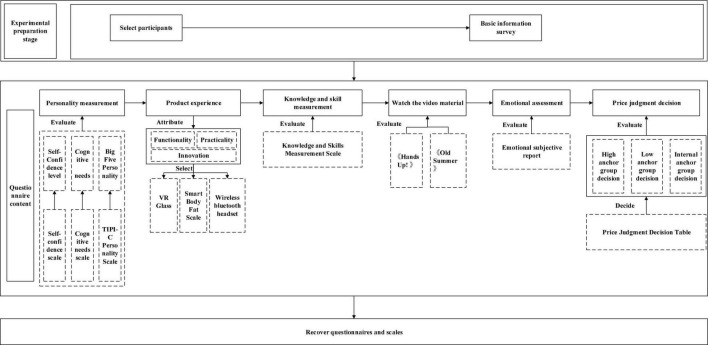
Flowchart of the formal experiment.

There was a total of 240 questionnaires, among which, 80 were from the high anchor group, 80 were from the low anchor group, and another 80 were from the internal anchor group; 120 included anchored warning indication, and 120 did not include anchored warning indication; and 120 were exerted time pressure and 120 had no time pressure. The flowchart of the formal experiment is shown in Figure 2.

## Experimental Results

### Existence and Influencing Factors of Anchoring Effect in the External Anchor Experiment

#### Existence of External Anchoring Effect

According to Formula (1), the anchoring effect index of the price judgment of the subjects was calculated. The results are shown in Table 3.

**TABLE 3:** Anchoring effect index of the external anchor group.

Questions	Q1	Q2	Q3
AI	0.784	0.818	0.477

The general range of anchoring effect index was 0–1, and the larger the value was, the stronger the anchoring effect would be. According to the statistical results, the subjects were influenced by anchoring effect on all the three questions, which suggested that consumers were affected by anchoring effect when making price judgment in a scene of external anchors. Thus, H1 was accepted.

#### Influencing Factors of External Anchoring Effect

##### Influence of Anchor Value on External Anchoring Effect

In order to explore the influence of the anchor value on anchoring effect under external anchor conditions, the experimental data of the high anchor decision-making group and the low anchor decision-making group were sorted out and the mean value and standard deviation of decision-making questions of the external anchor group were obtained (see Table 4).

**TABLE 4:** Mean value and standard deviation of decision-making questions of the external anchor group (M ± SD).

Questions	High anchor	Low anchor
Q1	0.5139 ± 0.0895	0.2053 ± 0.0951
Q2	0.0850 ± 0.0166	0.0343 ± 0.0092
Q3	0.1155 ± 0.0344	0.0880 ± 0.0210

Data analysis of Table 4 showed that the estimated value of price judgment by the subjects under the high anchor condition was obviously higher than that under the low anchor condition. Thus, when consumers made price judgment, they would adjust upward or downward with the reference value (anchor value) given by the outside world as a benchmark. A high anchor caused a high price judgment estimate, and a low anchor caused a low price estimate. Thus, H2 was accepted.

##### Anchoring Index of Influencing Factors Under External Anchor Conditions

According to Formula (2) and (3), the anchor indexes of gender, emotion, Big Five Personality, expert knowledge and skill, time pressure, early warning indication, cognitive need, and self-confidence level under high and low anchor conditions were obtained (see Table 5).

**TABLE 5:** Anchoring index of influencing factors under high and low anchor conditions (M ± SD).

Factors		Label	Assignment	Q1		Q2		Q3	
				High anchor	Low anchor	High anchor	Low anchor	High anchor	Low anchor
Gender		Male	1	0.542 ± 0.243	0.423 ± 0.277	0.532 ± 0.289	0.452 ± 0.253	0.555 ± 0.260	0.429 ± 0.197
		Female	2	0.614 ± 0.357	0.559 ± 0.244	0.644 ± 0.463	0.679 ± 0.357	0.691 ± 0.301	0.621 ± 0.240
Emotion		Positive	1	0.780 ± 0.693	0.636 ± 0.477	0.743 ± 0.384	0.529 ± 0.473	0.560 ± 0.329	0.499 ± 0.530
		Negative	2	0.655 ± 0.582	0.653 ± 0.439	0.631 ± 0.469	0.516 ± 0.556	0.465 ± 0.507	0.372 ± 0.375
Big Five Personality	Open	High	1a	0.639 ± 0.588	0.554 ± 0.433	1.239 ± 0.679	0.904 ± 0.584	0.854 ± 0.632	0.633 ± 0.677
		Low	1b	0.390 ± 0.532	0.377 ± 0.505	0.638 ± 0.545	0.503 ± 0.511	0.621 ± 0.533	0.438 ± 0.452
	Conscientious	High	2a	0.533 ± 0.461	0.744 ± 0.680	0.557 ± 0.335	0.530 ± 0.412	0.533 ± 0.529	0.402 ± 0.216
		Low	2b	0.945 ± 0.533	1.001 ± 0.576	0.714 ± 0.375	0.886 ± 0.371	0.646 ± 0.422	0.544 ± 0.340
	Extrovert	High	3a	0.899 ± 0.763	0.744 ± 0.404	0.901 ± 0.535	0.675 ± 0.531	0.639 ± 0.488	0.611 ± 0.293
		Low	3b	0.622 ± 0.400	0.438 ± 0.354	0.690 ± 0.332	0.607 ± 0.431	0.462 ± 0.256	0.425 ± 0.223
	Agreeable	High	4a	0.744 ± 0.737	0.721 ± 0.700	0.785 ± 0.579	0.744 ± 0.678	0.825 ± 0.661	0.744 ± 0.451
		Low	4b	0.637 ± 0.431	0.455 ± 0.265	0.535 ± 0.332	0.488 ± 0.423	0.803 ± 0.568	0.451 ± 0.459
	Neurotic	High	5a	0.643 ± 0.502	0.455 ± 0.509	0.698 ± 0.478	0.478 ± 0.364	0.489 ± 0.266	0.337 ± 0.401
		Low	5b	0.899 ± 0.599	0.721 ± 0.756	0.899 ± 0.619	0.750 ± 0.635	0.741 ± 0.501	0.711 ± 0.344
Expert knowledge and skill		More	1	0.455 ± 0.300	0.287 ± 0.277	0.412 ± 0.270	0.379 ± 0.359	0.420 ± 0.377	0.366 ± 0.305
		Less	2	0.935 ± 0.485	0.843 ± 0.769	1.143 ± 0.583	0.872 ± 0.553	0.755 ± 0.541	0.711 ± 0.539
Time pressure		With	1	0.380 ± 0.323	0.306 ± 0.231	0.282 ± 0.238	0.243 ± 0.249	0.279 ± 0.246	0.245 ± 0.177
		Without	2	0.271 ± 0.244	0.237 ± 0.351	0.362 ± 0.276	0.293 ± 0.162	0.384 ± 0.149	0.315 ± 0.254
Early warning indication		With	1	0.563 ± 0.355	0.435 ± 0.361	0.496 ± 0.427	0.468 ± 0.233	0.573 ± 0.460	0.513 ± 0.281
		Without	2	1.36 ± 0.623	0.809 ± 0.431	0.994 ± 0.677	0.841 ± 0.462	0.743 ± 0.580	0.639 ± 0.431
Cognitive need		High	1	0.482 ± 0.351	0.377 ± 0.382	0.428 ± 0.314	0.424 ± 0.309	0.346 ± 0.173	0.257 ± 0.205
		Low	2	0.679 ± 0.457	0.537 ± 0.209	0.798 ± 0.179	0.587 ± 0.201	0.500 ± 0.351	0.489 ± 0.266
Self-confidence level		High	1	0.577 ± 0.306	0.453 ± 0.316	0.566 ± 0.433	0.495 ± 0.377	0.416 ± 0.217	0.371 ± 0.306
		Low	2	1.37 ± 0.433	0.794 ± 0.506	0.981 ± 0.558	0.872 ± 0.297	0.710 ± 0.356	0.633 ± 0.274

From the above data, we can know that under external anchor conditions gender, emotion, Big Five Personality, knowledge, skill, and cognitive need influenced anchoring effect. Therefore, we supposed H3, H3a, H4, H5, H5a, H5b, H5c, H5d, H5e, H6, H6a, H9, and H9a were accepted. Although the subjects with time pressure and the subjects without time pressure were both influenced by anchoring effect under high and low anchor conditions, the degree of anchoring effect could not be evaluated, so H7 was rejected. The subjects with early warning indication were less affected by anchoring effect because they consciously avoided anchoring effect, while the subjects without early warning indication were strongly affected by anchoring effect. Therefore, no matter whether the early warning indication was set in the experiment, the subjects would both be affected by anchoring effect under external anchor conditions, but if there was no early warning indication, the anchoring effect would also be affected. Thus, H8 was only partially accepted. H10 and H10a were rejected because the data showed that subjects with a low self-confidence level showed a higher vulnerability to anchoring effect. The reason may be that subjects with a low self-confidence level relied too much on external anchoring information because it was difficult for them to make decisions on prices due to their sense of inferiority.

In general, the above factors had an anchoring effect under both high and low anchor conditions, and the anchoring effect under high anchor conditions was stronger than that under low anchor conditions. The reason may be that the subjects were stimulated by their questioning of the low anchor value caused by their price estimate lower than the actual product price under low anchor conditions to make in-depth reasoning and thinking in decision-making on the actual product price, making the final results more rational than that under high anchor conditions.

### Existence and Influencing Factors of Anchoring Effect in Internal Anchor Experiment

#### Existence of Internal Anchoring Effect

According to Formula (4), the mean skew index of the internal anchor group is shown in Table 6.

**TABLE 6:** Mean skew index of the internal anchor group.

Q	Actual value	Estimated average value	Estimated value boundary		Mean skew index
			Closest to anchor	Furthest away from anchor	
Q1	2999	2363	2850	1300	0.314
Q2	499	432	499	260	0.280
Q3	999	1020	900	650	0.480

The general range of mean skew index was 0–0.5. The smaller the value was, the stronger the anchoring effect would be. Table 6 showed that the mean skew indexes of the three questions were all less than 0.5, so consumers were affected by the internal anchoring effect when making price judgment. Thus, H1 was accepted. With further analysis, the mean skew index of Q1 and Q2 was smaller than that (0.480) of Q3, which indicated that anchoring effects of Q1 and Q2 were more significant.

#### Influencing Factors of Internal Anchoring Effect

##### Anchoring Index of Influencing Factors Under Internal Anchor Conditions

According to the calculation formula, respective mean skew indexes of influencing factors under internal anchor conditions were obtained. The mean skew indexes of the internal anchor group are shown in Table 7.

**TABLE 7:** Mean skew indexes of influencing factors under internal anchor conditions (M ± SD).

Factor		Label	Q1	Q2	Q3
Gender		Male	0.418 ± 0.089	0.442 ± 0.080	0.303 ± 0.137
		Female	0.311 ± 0.152	0.302 ± 0.132	0.218 ± 0.164
Emotion		Positive	0.358 ± 0.152	0.112 ± 0.134	0.426 ± 0.223
		Negative	0.413 ± 0.131	0.258 ± 0.117	0.446 ± 0.171
Big five personality	Open	High	0.285 ± 0.154	0.214 ± 0.151	0.312 ± 0.176
		Low	0.423 ± 0.107	0.387 ± 0.087	0.452 ± 0.133
	Conscientious	High	0.417 ± 0.101	0.403 ± 0.109	0.459 ± 0.141
		Low	0.319 ± 0.134	0.317 ± 0.135	0.419 ± 0.149
	Extrovert	High	0.112 ± 0.081	0.126 ± 0.101	0.412 ± 0.117
		Low	0.221 ± 0.371	0.334 ± 0.107	0.445 ± 0.085
	Agreeable	High	0.322 ± 0.108	0.350 ± 0.078	0.455 ± 0.092
		Low	0.211 ± 0.085	0.134 ± 0.088	0.439 ± 0.103
	Neurotic	High	0.456 ± 0.082	0.467 ± 0.080	0.470 ± 0.070
		Low	0.192 ± 0.107	0.236 ± 0.102	0.349 ± 0.098
Knowledge and skills		More	0.263 ± 0.281	0.294 ± 0.106	0.338 ± 0.179
		Less	0.381 ± 0.362	0.345 ± 0.086	0.392 ± 0.153
Time pressure		With	0.408 ± 0.173	0.379 ± 0.194	0.393 ± 0.193
		Without	0.396 ± 0.176	0.362 ± 0.127	0.471 ± 0.081
Early warning indication		With	0.195 ± 0.063	0.239 ± 0.097	0.204 ± 0.114
		Without	0.389 ± 0.183	0.292 ± 0.079	0.284 ± 0.145
Cognitive needs		High	0.392 ± 0.992	0.410 ± 0.992	0.497 ± 0.189
		Low	0.336 ± 0.740	0.383 ± 0.125	0.419 ± 0.161
Self-confidence level		High	0.481 ± 0.126	0.395 ± 0.108	0.406 ± 0.098
		Low	0.352 ± 0.160	0.274 ± 0.144	0.381 ± 0.139

From the above data, we can know that under external anchor conditions, gender, emotion, Big Five Personality, knowledge and skill, and cognitive need all influenced anchoring effect. Therefore, we supposed H3, H3a, H4, H5, H5a, H5b, H5c, H5d, H5e, H6, H6a, H8, H9, and H9a were accepted. Subjects with time pressure and subjects without time pressure showed little difference in their mean skew indexes under internal anchor conditions. Thus, H7 was rejected. Subjects with different self-confidence levels all showed an anchoring effect under internal anchor conditions. Thus, H10 was accepted. However, subjects with a low self-confidence level were significantly affected by the anchoring effect when making price judgment. Thus, H10a was rejected.

## Conclusion and Suggestions

### Conclusion

The above empirical results showed that the external anchoring effect and the internal anchoring effect existed simultaneously when consumers made price judgment in the experiencing scene. To be specific, under external anchor conditions, consumers made upward or downward adjustments to prices centering on anchor values. Gender, Big Five personality, expert knowledge and skill, early warning indication, cognitive need, and self-confidence level all had a significant impact on the anchoring effect. Particularly, when consumers made decisions on their familiar products, emotion and cognitive need had a significant impact on the anchoring effect, while time pressure had an insignificant impact on the anchoring effect.

### Recommendations

Being an important influencing factor of decision bias, anchoring effect provides a new idea for enterprise marketing planners. Based on the conclusions of this study, the following suggestions are put forward:

#### Use of External Anchor Conditions

##### Setting Reasonable Anchor Values to Induce the Purchase Intention of Consumers

In the process of experience marketing, enterprises can use external anchor conditions to create high and low anchor environment of products, such as setting the prices of the surrounding products or competitive products as references. By doing so, consumers can be subconsciously anchored when judging whether the current product price is reasonable or not, thus prompting their purchasing behaviors. In addition, the quality of anchors directly affects the anchoring effect. Besides selecting high anchor products as anchoring products, the prices of anchoring products must be transparent, credible, and authoritative. In this way, consumers can pay attention to the comparison between products and have more acceptance for anchors. Thus, their purchase intention can be triggered.

##### Focusing on Individual Difference and Designing Personalized Marketing Strategies

Because consumers of different genders and personalities have different processing methods in the comparison of anchored information, it is necessary to categorize consumers and design personalized marketing strategies. For example, giving male consumers concrete information that is comparable and highlighting the product’s own functions and service advantages; giving female consumers more distinctive innovative information, creating anchored environment, and improving female experience perception; and paying attention to mood swings of consumers of low openness and low extroversion personality in the process of product experience, and helping these consumers open their hearts by creating a relaxed experiencing atmosphere so as to be better anchored by the experiencing scene.

#### Use of Internal Anchor Conditions

##### Developing Differentiation Strategies and Reducing Sensitivity to Knowledge and Skill

Because of serious homogenization of market products, consumers accept a variety of information anytime and anywhere. Thus, their knowledge and skill are continuously enhanced. Therefore, enterprises should improve the competitive edge of their products by developing differentiation strategies to stay ahead of the knowledge and skill growth of consumers, so that consumers have no anchor to follow.

In the experience marketing mode, enterprises should improve the experience perception of consumers at the service level at any time, develop service differentiation strategies, create a good experience and shopping environment, and reduce consumers sensitivity to knowledge and skill through the internal anchor principle.

##### Highlighting Scenario Design and Leading Psychological Decision-Making

In an experience marketing mode, enterprises should be clever at creating an atmosphere to act in concert with anchors, for example, by designing music and short videos or decorating experience environment to bring consumers a sense of pleasure in terms of visual sense, hearing, smell, and touch so that consumers can be anchored by their pleasant experience process and experience perception to make psychological decisions that deviate from the actual prices.

## Limitations and Future Research Outlook

### Research Limitations

(1) Due to the time-consuming nature of the experiment and the complexity of the field experience, a total of 194 participants cooperated after the end of the formal experiment. The sample size is not rich enough, which may affect the universality of the experimental conclusions.

(2) There are many dimensions of experience situation, overall, including products, environment, services, and other dimensions. Due to the limitation of the experimental conditions, the participants mainly made price decisions based on the experience perception of the product itself (including product design concept, product characteristics, product practicality, product innovation function, etc.). The multi-dimension of the experience situation is not fully reflected, which may have a certain impact on the willingness of consumers to buy in the experience process.

(3) The research only studied the existence and influencing factors of anchoring effect under the specific scene of consumer experience. Due to the limited conditions, the psychological mechanism and neural mechanism behind the significant or insignificant influencing factors were not deeply explored, and it may not dig deep the factors that fully affect consumer price judgment.

### Outlook for Future Research

(1) At the theoretical level, the anchoring effect originated and developed in the West. The influencing factors and the paradigm of the anchor effect are all proposed by western scholars and widely used in the academic circle. Therefore, it is of great significance to deeply explore the influencing factors and interpretation mechanism of the marketing field anchor effect under the national conditions of China, which can promote the research on consumer purchase behavior and satisfaction under the sharing economy platform in the Internet era (Meilhan, 2019).

(2) In the network era, the traditional marketing method is no new idea for consumers. For businesses, they must stimulate the willingness of consumers to buy through new marketing perspectives, such as experience marketing, relationship marketing, and cultural marketing. Therefore, it is very necessary to study the existence and influencing factors of the anchor effect from a new perspective. It can provide new ideas for the pricing of business products and services, and explore new progress in the field of marketing. Especially in the sharing economy platform, reputation system is often used to purposefully play a supervisory role and effectively solve user privacy problems to build consumer trust on the platform and business (Hollowell et al., 2019; Popescu and Ciurlău, 2019).

(3) Anchoring effect comes from psychological theory, and consumers are quickly anchored largely because of the psychological changes of consumers in the decision-making process. Therefore, it is necessary to explore the logical reasons behind the factors of the anchor effect, especially in the field of price judgment. It is also necessary to conduct a deep exploration of the psychological and neural mechanisms that cause the anchor effect so as to promote the progress in this field. For example, the behavioral economics theory of decision-making, which explains consumer choice in the perspective of neural events, promotes the detection of the neural mechanisms that trigger consumer responses through neuroscience methods (Popescu et al., 2018).

## Data Availability Statement

The original contributions presented in the study are included in the article/supplementary material, further inquiries can be directed to the corresponding author/s.

## Ethics Statement

The studies involving human participants were reviewed and approved by the Tianjin University of Commerce. The patients/participants provided their written informed consent to participate in this study.

## Author Contributions

YZ and XG contributed to conception and design of the study. YZ organized the database, performed the statistical analysis, and wrote the first draft of the manuscript. XG wrote sections of the manuscript. Both authors contributed to manuscript revision, read, and approved the submitted version.

## Conflict of Interest

The authors declare that the research was conducted in the absence of any commercial or financial relationships that could be construed as a potential conflict of interest.

## Publisher’s Note

All claims expressed in this article are solely those of the authors and do not necessarily represent those of their affiliated organizations, or those of the publisher, the editors and the reviewers. Any product that may be evaluated in this article, or claim that may be made by its manufacturer, is not guaranteed or endorsed by the publisher.

## References

[B1] Abbott L. (1956). Quality and competition: an essay in economic theory. *Soc. Res.* 23 243–245. 10.7312/abbo92492

[B2] Bizzi L. Labban A. (2019). The double-edged impact of social media on online trading: opportunities, threats, and recommendations for organizations. *Bus. Horiz.* 62 509–519. 10.1016/j.bushor.2019.03.003

[B3] Bratu S. (2019). Can social media influencers shape corporate brand reputation? Online followers’ trust, value creation, and purchase intentions. *Rev. Contemp. Philos.* 18 157–163. 10.22381/RCP18201910

[B4] Briesch R. A. Krishnamurthi L. Mazumdar T. Raj S. P. (1997). A comparative analysis of reference price models. *J. Consum. Res.* 24 202–214. 10.1086/209505

[B5] Cacioppo J. T. Petty R. E. (1982). The need for cognition. *J. Pers. Soc. Psychol.* 42 116–131. 10.1037/0022-3514.42.1.116

[B6] Cacioppo J. T. Petty R. E. Kao C. F. (1984). The efficient assessment of need for cognition. *J. Pers. Assess.* 48 306–307. 10.1207/s15327752jpa4803_13 16367530

[B7] Chauvin B. Hermand D. Mullet E. (2010). Risk perception and personality facets. *Risk Anal.* 27 171–185. 10.1111/j.1539-6924.2006.00867.x 17362408

[B8] Chen J. Zhu H. H. (2019). Development and validity test of user experience scale from customer perspective. *Stat. Decis.* 35 60–64.

[B9] Chen N. Lu J. M. Wang H. B. (2014). Example anchoring effect in the process of interpersonal emotion prediction. *Psychol. Sci.* 37 930–935.

[B10] Chu Y. Q. (2020). Price anchor in mergers and acquisitions: financial innovation or value plunder- based on mergers and acquisitions of listed companies case analysis. *New Finance* 3 50–55.

[B11] Cooley D. Parks-Yancy R. (2019). The effect of social media on perceived information credibility and decision making. *J. Internet Commerce* 18 249–269. 10.1080/15332861.2019.1595362 31113480

[B12] Czerwonka M. (2017). Anchoring and overconfidence: the influence of culture and cognitive abilities. *Int. J. Manag. Econ.* 53 48–66. 10.1515/ijme-2017-0018

[B13] Disli M. Inghelbrecht K. Schoors K. Stieperaere H. (2020). Stock price anchoring. *SSRN Electron. J.* 1 1–28. 10.2139/ssrn.3749398

[B14] Englich B. Soder K. (2009). Moody experts: how mood and expertise influence judgmental anchoring. *Judgm. Decis. Mak.* 4 41–50.

[B15] Epley N. Gilovich T. (2001). Putting adjustment back in the anchoring and adjustment heuristic: differential processing of self-generated and experimenter-provided anchors. *Psychol. Sci.* 12 391–396. 10.1111/1467-9280.00372 11554672

[B16] Epley N. Gilovich T. (2006). The anchoring-and-adjustment heuristic: why the adjustments are insufficient. *Assoc. Psychol. Sci.* 19 311–320. 10.1111/j.1467-9280.2006.01704.x 16623688

[B17] Eroglu C. Croxton K. L. (2010). Biases in judgmental adjustments of statistical forecasts: the role of individual differences. *Int. J. Forecast.* 26 116–133. 10.1016/j.ijforecast.2009.02.005

[B18] Feng Z. Zheng L. L. (2019). Study of the gender differences in the college students’ online shopping irrational decisions-based on the anchoring effect theory. *J. Math. Pract. Theory* 49 145–152.

[B19] Furnham A. Boo H. C. (2011). A literature review of the anchoring effect. *J. Socio Econ.* 40 35–42. 10.1016/j.socec.2010.10.008

[B20] Gan P. C. (2019). Impact of customer experience on perceived value and customer loyalty. *Jiangsu Bus. Theory* 12 8–11.

[B21] Ge J. J. Li D. S. (2020). The big five personality and online knowledge payment behavior of university students. *J. Zhejiang Univ. Sci. Technol.* 32 393–400.

[B22] Gentile C. Spiller N. Noci G. (2007). How to sustain the customer experience: an overview of experience components that co-create value with the customers. *Eur. Manag. J.* 22 395–410. 10.1016/j.emj.2007.08.005

[B23] Gergaud O. Plantinga A. J. Ringeval-Deluze A. (2017). Anchored in the past: persistent price effects of obsolete vineyard ratings in France. *J. Econ. Behav. Organ.* 133 39–51. 10.1016/j.jebo.2016.10.005

[B24] Gross J. Levenson R. W. (1995). Emotion elicitation using films. *Cogn. Emot.* 9 87–108. 10.1080/02699939508408966

[B25] Hollowell J. C. Rowland Z. Kliestik T. Kliestikova J. Dengov V. V. (2019). Customer loyalty in the sharing economy platforms: how digital personal reputation and feedback systems facilitate interaction and trust between strangers. *J. Self Govern. Manag. Econ.* 7 13–18. 10.22381/JSME7120192

[B26] Holst G. S. Hermann D. Musshoff O. (2015). Anchoring effects in an experimental auction-Are farmers anchored? *J. Econ. Psychol.* 48 106–117. 10.1016/j.joep.2015.03.008

[B27] Jacowitz K. E. Kahneman D. (1995). Measures of anchoring in estimation tasks. *Pers. Soc. Psychol. Bull.* 21 1161–1166. 10.1177/01461672952111004

[B28] John O. P. Srivastava S. (1999). “The big five trait taxonomy: history, measurement, and theoretical perspectives,” in *Handbook of Personality: Theory and Research*, eds Pervin L. A. John O. P. (New York, NY: Guilford), 102–138.

[B29] Kahneman D. Tversky A. (1979). Prospect theory: an analysis of decisions under risk. *Econometrica* 47 263–292. 10.2307/1914185

[B30] Koçaş C. Dogerlioglu-Demir K. (2020). The 1 in 1,000,000: context effects of how numbers cue different kinds of incidental environmental anchoring in marketing communications. *J. Bus. Res.* 109 536–544. 10.1016/j.jbusres.2019.01.027

[B31] Lauriola M. Levin I. P. (2001). Relating individual differences in attitude toward Ambiguity to risky choices. *J. Behav. Decis. Mak.* 14 107–122. 10.1002/bdm.368

[B32] Li L. Z. Maniadis Z. Sedikides C. (2021). Anchoring in economics: a meta-analysis of studies on willingness-to-pay and willingness-to-accept. *J. Behav. Exp. Econ.* 90:101629. 10.1016/j.socec.2020.101629

[B33] Li Z. (2019). Who created the experience? The three models of experience creation and their operating mechanisms. *Nankai Bus. Rev.* 22 178–191.

[B34] Luo Q. Chen J. K. (2019). Study on the influencing factor of consumer’s willingness to consume in the New Retail Business ——Based on the view of the customer’s experience. *Econ. Res. Guide* 8 116–121.

[B35] Mcelroy T. Dowd K. (2007). Susceptibility to anchoring effects: how openness-to-experience influences responses to anchoring cues. *Judgm. Decis. Mak.* 2 48–53.

[B36] Meilhan D. (2019). Customer value co-creation behavior in the online platform economy. *J. Self Govern. Manag. Econ.* 7 19–24. 10.22381/JSME7120193

[B37] Mircică N. (2020). Restoring public trust in digital platform operations: machine learning algorithmic structuring of social media content. *Rev. Contemp. Philos.* 19 85–91. 10.22381/RCP1920209

[B38] Mirică (Dumitrescu) C.-O. (2019). The behavioral economics of decision making: explaining consumer choice in terms of neural events. *Econ. Manag. Financ. Mark.* 14 16–20. 10.22381/EMFM14120192

[B39] Mussweiler T. Englich B. (2005). Subliminal anchoring: judgmental consequences and underlying mechanisms. *Organ. Behav. Hum. Decis. Process.* 98 133–143. 10.1016/j.obhdp.2004.12.002

[B40] Popescu G. H. Ciurlău F. C. (2019). Making decisions in collaborative consumption: digital trust and reputation systems in the sharing economy. *J. Self Govern. Manag. Econ.* 7 7–12. 10.22381/JSME7120191

[B41] Popescu G. H. Petrescu I. E. Sabie O. M. Muşat M. (2018). Labor displacing technological change and worldwide economic insecurity: how automation and the creation of innovative tasks shape inequality. *Psycho Sociol. Issues Hum. Resour. Manag.* 6 80–85. 10.22381/PIHRM6220188

[B42] Pour M. J. Taheri F. (2019). Personality traits and knowledge sharing behavior in social media: mediating role of trust and subjective wellbeing. *Horizon* 27 98–117. 10.1108/OTH-03-2019-0012

[B43] Reitsma-van Rooijen M. Daamen D. D. (2006). Subliminal anchoring: the effects of subliminally presented numbers on probability estimates. *J. Exp. Soc. Psychol.* 42 380–387. 10.1016/j.jesp.2005.05.001

[B44] Schwarz N. (2002). “Feelings as information: moods influence judgments and processing strategies,” in *Heuristics and Biases: The Psychology of Intuitive Judgment*, eds Gilovich T. Griffin D. Kahneman D. (Cambridge, MA: Cambridge University Press), 534–547. 10.1017/CBO9780511808098.031

[B45] Shen C. H. Cheng F. Wei C. X. (2016). Research on the relationship between anchoring effect and consumer purchase intention. *Consum. Econ.* 32 57–63.

[B46] Simonson I. Drolet A. (2004). Anchoring effects on consumers’ willingness-to-pay and willingness-to-accept. *J. Consum. Res.* 31 681–690. 10.1086/425103

[B47] Song J. H. (2019). Inquiry into the adjustment and utilization of the anchoring effect mechanism in criminal trials. *Legal Syst. Soc.* 4 99–100.

[B48] Su B. W. Hu Q. L. Wang F. (2019). A comparative study on the customer experience of supermarkets under the new retail background-based on Ahp-Pca. *J. Baoding Univ.* 32 68–72.

[B49] Toffler A. (1970). *Future Shock.* New York, NY: Bantam Books, Inc.

[B50] Tong Y. Q. Yang J. Zhao Z. Y. (2020). Exploration on the customer experience value factors of Lvshun museum. *Packag. Eng.* 41 147–153.

[B51] Tversky A. Kahneman D. (1974). Judgment under uncertainty: heuristics and biases. *Science* 185 1124–1131. 10.1126/science.185.4157.1124 17835457

[B52] Von Hecker U. Klauer K. C. Aßfalg A. (2019). A robust anchoring effect in linear ordering. *Q. J. Exp. Psychol.* 72 2680–2689. 10.1177/1747021819855234 31104594

[B53] Wang R. J. Zhu Y. Wang J. J. (2021). The impact of clothing cross-border consumption experience on brand assets and willingness to buy. *Silk* 58 40–46.

[B54] Wang X. L. (2013). Discuss the anchoring effect of heuristic bias in decision-making. *Business* 19 146–147.

[B55] Weng Q. X. Peng C. H. Cao W. L. Xi Y. M. (2016). The relationship between big five personality and subjective career success: a meta-analysis of research in the Past 15 years. *Manag. Rev.* 28 83–95.

[B56] Xu Q. (2015). A review of research on anchoring effects at home and abroad. *Coop. Econ. Technol.* 20 94–95.

[B57] Yan X. P. Li Y. X. Liu P. N. (2021). Magnetic anchoring technique assisted thoracoscopic lung resection clinical application effect. *J. Xi’an Jiaotong Univ.* 42 262–266.

[B58] Yang J. H. (2015). An empirical study on the impact of consumer experience on retailers’ brand assets. *Jinan J.* 37 38–47.

[B59] Yang X. D. (2017). The influence of price setting factors on consumers’ purchase intention—based on the Internet pre-sale environment. *Bus. Econ. Res.* 18 30–32.

[B60] Yao Y. H. Yu Q. Weng J. H. Li P. R. Huang G. L. (2021). Research on 50ETF option anchoring effect. *Operat. Manag.* 3 18–22.

[B61] Yoon S. Fong N. (2019). Uninformative anchors have persistent effects on valuation judgments. *J. Consum. Psychol.* 29 391–410. 10.1002/jcpy.1091

[B62] Yu L. Q. Gao Z. F. Sims C. A. Guan Z. F. (2017). “Effect of price on consumers’ willingness to pay: is it from quality perception or price anchoring?,” in *Proceedings of the 2017 Agricultural & Applied Economics Association Annual Meeting*, (Chicago, IL: Agricultural and Applied Economics Association).

[B63] Yuan G. (2013). Discuss the application of anchoring effect in enterprise marketing from controllable factors. *China Market* 41 48–51.

[B64] Zhang S. X. Dong J. C. (2019). Anchoring effect in accounting professional judgment and decision making. *Commun. Financ. Account.* 25 7–12.

[B65] Zhang Y. T. Liu Y. L. (2021). Cause and governance research of the “anchor effect” of Chinese fund investors. *Economist* 8 67–92.

[B66] Zheng L. M. (2015). Anchoring effect of the price judgment estimate and its experimental study. *Mod. Manag. Sci.* 12, 64–66.

[B67] Zheng P. Liu C. H. Yu G. L. (2012). A review of emotion induction methods. *Prog. Psychol. Sci.* 20 45–55. 10.3724/SP.J.1042.2012.00045

[B68] Zou D. Q. Wang G. Zhao P. Wang Y. (2007). The impact of functional value and symbolic value on brand loyalty: the moderating effect of gender differences and brand differences. *Nankai Manag. Rev.* 10 4–12.

